# Effects of an integrated neighborhood approach on older people’s (health-related) quality of life and well-being

**DOI:** 10.1186/s13104-016-2254-5

**Published:** 2016-09-23

**Authors:** Hanna M. van Dijk, Jane M. Cramm, Erwin Birnie, Anna P. Nieboer

**Affiliations:** Institute of Health Policy and Management, Erasmus University Rotterdam, Burgemeester Oudlaan 50, 3000 DR Rotterdam, The Netherlands

**Keywords:** Integrated neighborhood approach, Effects, Quasi-experimental study, Community-dwelling older people

## Abstract

**Background:**

Integrated neighborhood approaches (INAs) are increasingly advocated to reinforce formal and informal community networks and support community-dwelling older people. They aim to augment older people’s self-management abilities and engage informal networks before seeking professional support. INAs’ effectiveness however remains unknown. We evaluated an INA’s effects on older people’s (health-related) quality of life (HRQoL) and well-being in Rotterdam.

**Methods:**

We used a matched quasi-experimental design comparing INA with “usual” care and support. Community-dwelling frail older (70+ years) people and frailty- and gender-matched control subjects (n = 186 each) were followed over a 1-year period (measurements at baseline and 6 and 12 months). Primary outcomes were HRQoL (EQ-5D-3L, SF-20) and well-being [social production function instrument for the level of well-being (SPF-IL)]. The effect of INA was analysed using an “intention to treat” and an “as treated” approach.

**Results:**

The results indicated that pre-intervention participants had lower incomes and were significantly older, more often single, less educated and more likely to have ≥1 disease than control subjects; they had lower well-being, physical functioning, role functioning, and mental health. Generalized linear mixed modelling of repeated measurements revealed no substantial difference in well-being or HRQoL between the intervention and control group after 1 year. The small differences we did find in the intention to treat group though were in favour of the control subjects (SF-20 = 6.98, 95 % confidence interval [CI] = 2.45–11.52; SPF-IL = .09, 95 % CI = .01–.17). However, the difference in well-being (SPF-IL) disappeared in the as treated analysis.

**Conclusions:**

The lack of effects of INA highlights the complexity of integrated care and support initiatives. Barriers associated with meeting the complex, varied needs of frail older people, and those related to dynamic political and social climates challenge initiative effectiveness.

*Trial registration* The research was supported with a grant provided by the Netherlands Organisation for Health Research and Development (ZonMw, project number 314030201) as part of the National Care for the Elderly Programme

## Background

Integrated neighborhood approaches (INAs) are increasingly advocated as means to create a supportive environment for the growing number of community-dwelling older people with (complex) needs [1–3]. INAs, consisting of collaboration among municipalities, health and social care providers, and informal care, aim to integrate available neighborhood resources and increase responsiveness to citizens’ specific needs [1, 4]. Although the need for INAs to achieve a better balance between support of increasing numbers of care-dependent older people in the community and protection of their (health-related) quality of life is widely recognized, the effectiveness of such programs is currently unknown.

In 2011, the Rotterdam municipality, local health and social care organizations, Erasmus University Rotterdam, the University of Applied Sciences, and Geriatric Network Rotterdam initiated an INA for community-dwelling older people. Its overarching aim was to create a supportive environment allowing community-dwelling older people to live independently. The INA aims to overcome barriers associated with the provision of care and support in the Netherlands, which is often characterized as reactive, i.e., lacking a proactive and preventive approach that aims to protect older people’s (health-related) quality of life, and fragmented, i.e., lacking a coordinated approach to health and social care service provision. In the Netherlands, general practitioners (GPs) play a gatekeeper role in *health care* service provision, referring (older) patients to primary, secondary, or tertiary health care professionals when necessary [5]. Municipalities assume responsibility for *social* services, such as household services and support for informal caregivers. Older people can apply for these welfare services, and their eligibility is assessed based on their needs and capabilities [6, 7]. Only when care and support cannot be provided for by older people themselves or their informal network for objective reasons, such as insufficient economic means and/or the absence of informal caregivers, do municipalities have a mandatory responsibility to compensate for older people’s limitations in various areas, such as transport or household support.

Currently, collaboration and resource integration among health and social care providers and informal support givers is insufficient to support the ability of community-dwelling older people to age in place [8]. Thus, the INA combines components found to be effective for integrated care and support provision, such as the integration of health and social care services, a demand-driven and person-centered approach, the use of multidisciplinary and outreach teams, and preventive home visits [2, 9–13]. The INA also incorporates increasingly promoted innovative components, such as the engagement of informal caregivers and the community and the strengthening of self-management abilities [8]. By reinforcing networks among health and social care providers and informal support givers in the community, formal and informal support givers become mutually responsible for optimizing current services and supporting older people’s ability to age in place. This may foster early recognition of older people’s needs and encourage effective self-management, which may both positively influence older people’s (health-related) quality of life and well-being. This enables the older people to ‘star’ in the ‘production’ of their own well-being as a form of empowerment.

In this study, we evaluated the INA’s effects on older people’s (health-related) quality of life and well-being. To our knowledge, this study is the first to evaluate an INA’s effects; it thus provides valuable insight into whether INAs can meet expectations by contributing to the (health-related) quality of life and well-being of community-dwelling older people.

## Methods

### Study design and inclusion

We used a matched quasi-experimental design to compare outcomes of older people who participated in the INA and those who received “usual” care and support. Measurements were taken at baseline (T0; pre-intervention) and at 6 (T1) and 12 months (T2). Older people (a) aged 70 or more years who (b) lived independently (i.e., not in an institutional setting) in one of four INA neighborhoods in Rotterdam (Lage Land/Prinsenland, Lombardijen, Oude Westen, and Vreewijk), (c) were frail, and (d) consented to study participation were eligible for inclusion. Frailty was assessed using the Tilburg Frailty Indicator (TFI), a multidimensional instrument that captures physical, psychological, and social domains of frailty [6].

Intervention group members were recruited by community workers, who engaged other professionals and community members in reporting signals of frailty. After identifying potentially frail older people, community workers visited them at home and administered the TFI during the first or second home visit. Older people in the intervention group were matched 1:1 with control subjects on the basis of TFI score (≥5) and gender. We recruited control subjects by sending questionnaires to a random sample of community-dwelling older people residing in neighborhoods with socioeconomic characteristics comparable to those of INA neighborhoods. The questionnaire included the TFI instrument for matching purposes. Among respondents, we identified older people who matched intervention subjects according to TFI score and gender, and randomly invited subjects by telephone to participate in the study.

The project and evaluation are part of the National Care for the Elderly Programme, launched in 2008 and funded by the Netherlands Organization for Health Research and Development (Project No. 314030201). The ethics committee of Erasmus University Medical Center, Rotterdam, the Netherlands, approved the project in June 2011 (MEC-2011-197). Written informed consent was obtained from all participating respondents. Since the assignment of the intervention was not under the discretion of the investigators, we did not need to register our trial.

### Intervention

**Fig. 1:**
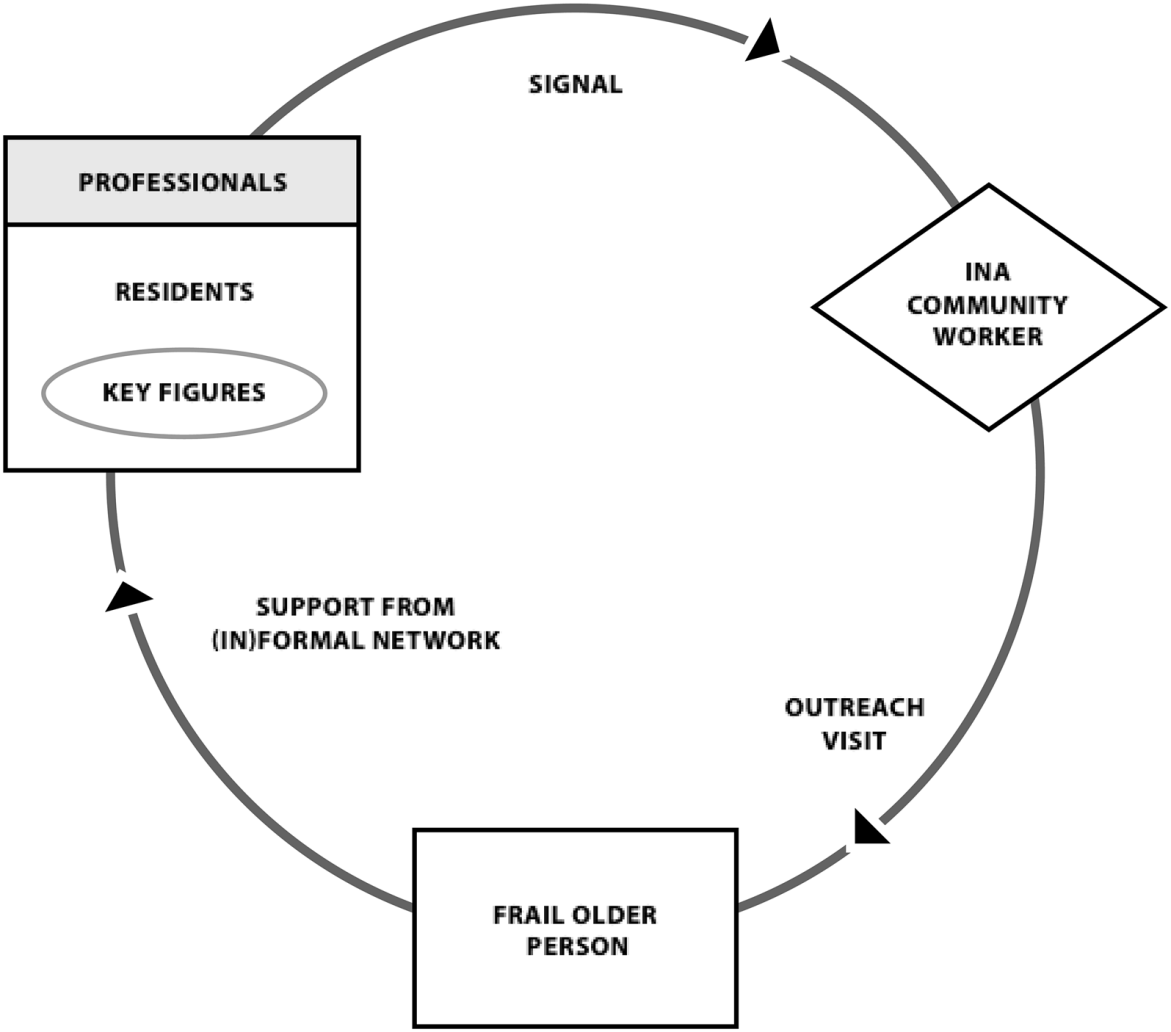
Working method of the integrated neighborhood approach (INA)

The INA was initiated in April 2011 in two Rotterdam neighborhoods and extended to two additional neighborhoods 1 year later. Within the context of the INA, professionals and residents are asked to watch over neighbors and report manifestations of frailty to INA community workers (Fig. 1).

Community workers had health and social care backgrounds and were temporarily reassigned to INA teams, many of which included at least one social worker and community nurse familiar with the neighborhood. Community workers visited older people at home and mapped their social and physical needs and capabilities with respect to factors such as housing, mobility issues, and social activities, through phased interviews. Together with older people, they sought appropriate solutions to identified problems or needs and composed individualized support plans. First, community workers assessed older people’s capabilities and self-management abilities and sought to increase their responsibility for their health and well-being, for example when applying for a walker or learning to manage finances. For older people who could not meet their own needs, INA community workers sought informal interventions, e.g., finding a neighbor willing to bring groceries or setting up an activity with the help of neighbors, before relying on professional support. Community workers thus served as liaisons at the personal (supporting and monitoring older people), professional (seeking a multidisciplinary approach to support), and community (establishing a well-functioning network and engaging informal support givers) levels. Few guidelines were set for INA community workers performing these roles and accompanying tasks, giving them professional autonomy to create their own working methods. Box 1 describes a real-life case illustrating the INA approach. Further details of the INA’s scope, aims, and study protocol have been published elsewhere [8].

**Box 1:** Real-life case of an INA participant in Rotterdam

*Mrs. Schols, a 75-year-old woman, resides in a large apartment block in a Rotterdam suburb. She has no children and has lived alone since her husband’s passing 10 years ago. Mrs. Schols used to enjoy working as a receptionist in the banking sector, but was forced to quit due to lung disease (COPD). This disease had major impacts not only on her working life, but also on her social life. Apart from receiving personal assistance and home care, Mrs. Schols is being monitored by a kind next-door neighbor, Mr. Markus. For some time, Mr. Markus has noticed that Mrs. Schols comes outside only occasionally, leaving him worried about her physical condition. He also wonders whether Mrs. Schols might be entitled to more amenities due to her physical decline. * *Through the neighborhood center, Mr. Markus meets an INA community worker. After hearing his concerns about Mrs. Schols, the community worker schedules a home visit to gain further insight into her needs. This visit soon reveals that Mrs. Schols does not have increasing physical needs, as Mr. Markus had suggested, but rather a growing social need due to her shrinking social network. She misses having someone to talk to about her disease and longs for someone who is willing to take a walk with her. Due to her fear of riding her mobility scooter, especially given that she must carry an oxygen tank, she is hesitant to go outdoors. * *After the home visit, the community worker seeks someone who would be willing to support Mrs. Schols. Through an advertisement in the local newspaper, she soon finds an enthusiastic nearby neighbor. When the two meet, they immediately get along. Currently, the neighbor visits Mrs. Schols every week and walks with her or takes her to the supermarket to buy groceries. She also helps Mrs. Schols practice with her mobility scooter, enabling her to go outside by herself*	

### Instruments and data collection

The primary outcomes were (health-related) quality of life and well-being. The validated five-dimensional, three-level EuroQol instrument (EQ-5D-3L) was administered to describe older people’s health-related quality of life in terms of mobility, self-care, usual activities, pain/discomfort, and anxiety/depression [14]. Preference weights were assigned to the resulting health profiles to obtain summary valuations or utility scores, with 1 representing the utility of best imaginable health state, 0 representing death or a health state considered to be equivalent to death, and negative values indicating health states considered to be worse than death [15]. We used five subscales of the validated Dutch version of the Short Form-20 (SF-20) to assess the following dimensions of generic (health-related) quality of life: physical functioning, role functioning, social functioning, mental health, and health perceptions [16, 17]. All scales were transformed to range from 0 to 100, with higher scores indicating better functioning. Finally, we used the 15-item version of the social production function instrument for the level of well-being (SPF-IL) scale [18] to assess respondents’ ability to meet the universal goals needed to enhance subjective well-being: affection, behavioral confirmation, status, comfort, and stimulation. Mean scores range from 1 to 4, with higher scores indicating greater well-being.

To enhance data quality and minimize missing values and study drop out, trained interviewers administered the questionnaires during home visits. Average interview length was about 90 min. Intervention and control participants were rewarded with incentives (a cookie jar at T0, a notepad with pencil at T1, and a card game at T2).

Besides administering questionnaires among older people, INA’s community workers filled in individualized support plans with information on the support-giving process. To establish the intention to treat vs. as treated group, we conducted a file audit of these support plans to assess whether older people received any intervention, i.e. whether INA’s community workers arranged (in)formal support; not the intensity of the support that was provided. The support plans of 18 older people revealed that no intervention was provided (often because older people felt not in need of support or felt reluctant about receiving support); therefore, these cases were removed in the as treated analysis.

### Sample size

Given the anticipated 27 % drop-out rate between T0 and T2 (e.g., due to death, moving, nursing home admission, or no longer wishing to participate) [12] we aimed to include 186 older people each in the intervention and control group. This sample size was based on a pilot study of frail older people in the control neighborhoods and was required to detect a .16-point (=1/3 standard deviation [SD]) improvement in SPF-IL score in the intervention group compared with the control group at T2 (based on a mean SPF-IL-score of 2.42 [SD = .47]; alpha (two-sided) = .05, beta = .10). This sample size was also sufficient to detect improvements in other outcome measures.

**Fig. 2:**
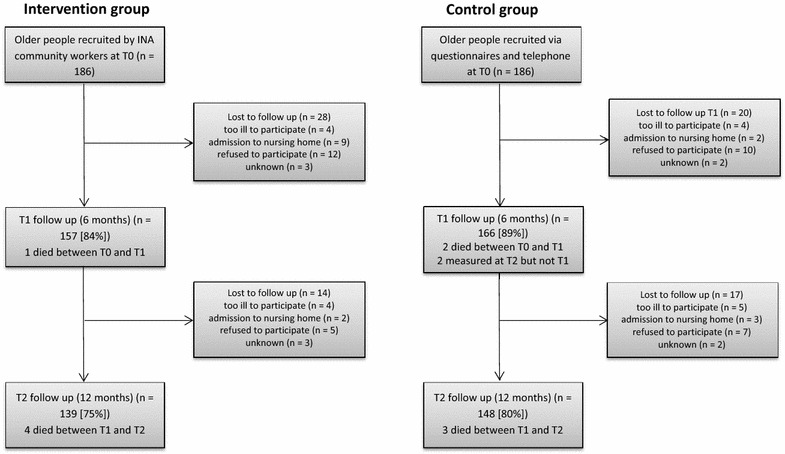
Flow chart of study participation of the integrated neighborhood approach (INA)

As illustrated by Fig. 2, we were able to include a sufficient number of participants. At baseline, 372 intervention and control subjects (n = 186 each) were recruited. Observations were available for 323 (87 %) participants at T1 and 287 (78 %) participants at T2. Measurements from all three timepoints were available for 285 (77 %) participants.

### Statistical methods

Baseline differences between groups were assessed using unpaired Student’s *t*-tests for continuous variables with (approximately) normal distributions, Mann–Whitney *U*-tests for continuous variables with non-normal distributions, and Chi-squared tests for categorical variables. Intervention effectiveness was examined using unadjusted (i.e., excluding adjusting covariables, but including time) and adjusted comparisons. We performed an intention to treat analysis as well as an as treated analysis in which older people were analyzed according to the actual intervention received. Those that did not receive any intervention (n = 18) were excluded from the as treated analysis. We used general linear mixed models of repeated measurements to analyze differences in outcomes between groups (covariance type: unstructured; beta distribution; all fixed effects). Dependent variables were EQ-5D-3L, SPF-IL, and SF-20 scores. Independent variables were baseline scores of the studied outcome variables; gender and frailty (TFI score) as matching factors; time and intervention/control group as main effects; and age, educational and income levels (low or high), living situation (single or not), and morbidity (0, 1, or >1 disease) as adjusting covariables. Additional analyses were performed to determine interactions between time and group membership, as well as the influence of neighborhood level on outcomes; these analyses revealed no other effect on any outcome and their results are not presented. Goodness of fit was expressed using the –2 log likelihood and Akaike’s information criterion, with lower scores indicating better fit. *p* < .05 (two-sided) was considered to indicate a statistically significant difference. SPSS was used for all statistical analyses.

## Results

### Baseline characteristics

Table 1 shows participants’ baseline characteristics. At T0, compared with control subjects, participants in the intervention group (both intention to treat and as treated participants) were significantly older, more often single, and less educated; they had lower incomes, were more likely to have one or more diseases, and had lower SPF-IL scores and lower SF-20 scores for the physical functioning, role functioning, and mental health dimensions. No significant difference in health-related quality of life (EQ-5D-3L score), SF-20 social functioning or current health perceptions score was observed.

**Table 1:** Baseline characteristics of older people

	Control group	Intention to treat^a^	As treated^a^
	n = 186	n = 186	n = 168
Age (years)	79.8 (5.9)	81.6 (6.0)**	81.6 (6.0)**
Sex (female)	137 (73.7 %)	143 (67.9 %)	127 (75.6 %)
Living situation (single)	153 (82.3 %)	167 (89.9 %)*	152 (90.5 %)*
Educational level (low)	37 (19.9 %)	73 (39.2 %)***	65 (38.7 %)***
Income (low)	99 (53.2 %)	124 (66.7 %)**	113 (67.3 %)**
Morbidity (≥1 disease)	182 (97.8 %)	173 (93.0 %)*	157 (93.5 %)*
Frailty (TFI)	8.0 (2.2)	8.1 (2.3)	8.2 (2.3)
Well-being (SPF-IL)	2.7 (.43)	2.6 (.56)**	2.6 (.55)**
Health-related quality of life (EQ-5D-3L)	.69 (.25)	66 (.26)	.65 (.26)
SF-20 physical functioning	45.1 (30.4)	38.3 (31.6)*	38.2 (31.8)*
SF-20 role functioning	31.6 (42.5)	23.4 (36.4)*	23.8 (36.6)*
SF-20 social functioning	65.3 (32.2)	60.2 (37.2)	59.6 (37.1)
SF-20 mental health	67.3 (21.8)	61.6 (24.2)*	61.3 (24.2)*
SF-20 current health perceptions	45.6 (9.8)	47.1 (9.3)	47.0 (9.5)

Values are presented as mean (standard deviation) or n (%)

*TFI* Tilburg frailty indicator, *SPF-IL* social production function instrument for the level of well-being, *EQ-5D-3L* five-dimensional, three-level EuroQol, *SF-20* short form 20

* *p* ≤ .05 (two-tailed)

** *p* ≤ .01

*** *p* ≤ .001

^a^Statistics compared to control group

### One-year changes in well-being and (health-related) quality of life

No substantial difference in well-being or (health-related) quality of life was observed between the intervention and control group at 1 year (T2) in analyses adjusted for time, age, sex, educational level, income, living situation, morbidity, frailty, and baseline scores. Control group participants (Table 2) reported better physical functioning (SF-20 dimension score = 6.98, 95 % confidence interval [CI] = 2.45–11.52) and well-being (SPF-IL score = .09, 95 % CI = .01–.17) than did intention to treat participants at 1 year. However, as treated analysis (Table 3) revealed no significant difference in well-being (SPF-IL score = .07, 95 % CI = −.01 to .15). Last, it is worth notifying that differences favoring the control group at baseline on role functioning and mental health disappear at 1 year in both the intention to treat and as-treated analyses.

**Table 2:** Generalized linear mixed modeling of outcomes: intention to treat analysis (differences between groups over time)

	Unadjusted overall effects^a^						Adjusted overall effects^b^					
	Mean (SE) control	Mean (SE) intervention	Mean difference (95 % CI)	p	–2 log likelihood	AIC	Mean (SE) control	Mean (SE) intervention	Mean difference (95 % CI)	*p*	–2 log likelihood	AIC
Well-being (SPF-IL)	2.65 (.04)	2.47 (.04)	–.18 (–.28 to –.08)	<.001	673.799	679.799	2.50 (.60)	2.41 (.60)	–.09 (–.17 to –.01)	.031	509.924	515.924
Health-related quality of life (EQ-5D-3L)	.77 (.01)	.73 (.01)	–.04 (–.08 to –.01)	.014	–538.810	–532.810	.77 (.02)	.75 (.02)	–.02 (–.05 to .01)	.121	−647.945	−641.945
*Quality of life (SF-20)*												
Physical functioning	45.73 (2.32)	33.73 (2.38)	–12.00 (–18.53 to –5.47)	<.001	5766.759	5772.759	44.21 (3.27)	37.22 (3.22)	–6.98 (–11.52 to –2.45)	.003	5473.313	5479.313
Role functioning	32.62 (2.87)	22.70 (2.95)	–9.92 (–18.00 to –1.83)	.016	6117.607	6123.607	36.44 (5.11)	30.63 (5.03)	–5.81 (–12.87 to 1.24)	.106	5962.798	5968.798
Social functioning	64.02 (2.26)	60.45 (2.33)	–3.57 (–9.93 to 2.80)	.272	5884.469	5890.469	64.16 (4.07)	64.00 (4.04)	–.15 (–5.71 to 5.40)	.957	5673.431	5679.431
Mental health	66.19 (1.49)	61.45 (1.53)	–4.74 (–8.93 to –.55)	.027	5285.270	5291.270	66.98 (2.29)	65.20 (2.26)	–1.77 (–4.96 to 1.42)	.276	5050.625	5056.625
Current health perceptions	46.58 (.55)	47.06 (.56)	.48 (–1.06 to 2.02)	.540	4329.900	4335.900	45.99 (1.12)	46.70 (1.10)	.71 (–.81 to 2.24)	.359	4276.356	4282.356

*SE* standard error of the mean, *CI* confidence interval, *AIC* Akaike’s information criterion, *SPF-IL* social production function instrument for the level of well-being, *EQ-5D-3L* five-dimensional, three-level EuroQol, *SF-20* short form 20

^a^Excluding adjusting covariables, but including time

^b^Adjusted for age, sex, education, income, living situation, morbidity, frailty, time and baseline scores of studied outcomes

**Table 3:** Generalized linear mixed modeling of outcomes: as treated analysis (differences between groups over time)

	Unadjusted overall effects^a^						Adjusted overall effects^b^					
	Mean (SE) control	Mean (SE) intervention	Mean difference (95 % CI)	p	–2 log likelihood	AIC	Mean (SE) control	Mean (SE) intervention	Mean difference (95 % CI)	*p*	–2 log likelihood	AIC
Well-being (SPF-IL)	2.65 (.04)	2.46 (.04)	–.19 (–.29 to –.09)	<.001	640.287	646.287	2.48 (.60)	2.41 (.60)	–.07 (–.15 to .01)	.092	477.725	483.725
Health-related quality of life (EQ-5D-3L)	.77 (.01)	.72 (.01)	–.05 (–.08 to –.01)	.010	−510.963	−504.963	.77 (.02)	.74 (.02)	–.02 (–.05 to .01)	.142	−510.963	−504.963
*Quality of life (SF-20)*												
Physical functioning	45.69 (2.34)	33.16 (2.51)	–12.54 (–19.28 to 5.79)	<.001	5510.560	5516.560	44.00 (3.42)	37.18 (3.43)	–6.82 (–11.50 to 2.15)	.004	5510.560	5516.560
Role functioning	32.60 (2.91)	23.41 (3.13)	–9.20 (–17.59 to –.80)	.032	5850.705	5856.705	37.75 (5.36)	33.04 (5.37)	–4.72 (–12.01 to 2.58)	.204	5850.705	5856.705
Social functioning	64.00 (2.26)	59.71 (2.44)	–4.29 (–10.82 to 2.25)	.198	5618.888	5624.888	61.55 (4.28)	61.60 (4.31)	.44 (–5.68 to 5.76)	.988	5618.888	5624.888
Mental health	66.19 (1.47)	60.93 (1.58)	−5.26 (−9.51 to −1.01)	.015	5032.019	5038.019	65.89 (2.39)	64.28 (2.40)	–1.61 (–4.88 to 1.66)	.334	5032.019	5038.019
Current health perceptions	46.58 (.55)	46.86 (.59)	.27 (–1.31 to 1.86)	.733	4145.909	4151.909	45.81 (1.17)	46.49 (1.18)	.67 (–.90 to 2.25)	.401	4145.909	4151.909

*SE* standard error of the mean, *CI* confidence interval, *AIC* Akaike’s information criterion, SPF-IL social production function instrument for the level of well-being, *EQ-5D-3L* five-dimensional, three-level EuroQol, *SF-20* short form 20

^a^Excluding adjusting covariables, but including time

^b^Adjusted for age, sex, education, income, living situation, morbidity, frailty, time and baseline scores of studied outcomes

## Discussion

INAs are increasingly advocated to support community-dwelling older people, but their effectiveness has not been examined previously. This study thus assessed the effectiveness of an INA using measures of older people’s (health-related) quality of life and well-being. The INA was found to have no substantial effect; the control group showed slightly better well-being and physical functioning than the intention to treat group, but these differences were not clinically relevant. The minimal clinically relevant difference in these cases would be .5 SD [19] or equivalently .28 for well-being and 15.08 for physical functioning, whereas our study showed effect sizes of .09 and 6.98 respectively.

Furthermore, differences in well-being disappeared in the as treated analysis. Last, the slightly positive result was found that differences favoring the control group at baseline on role functioning and mental health disappear at 1 year in both the intention to treat and as-treated analyses. However, it remains unclear whether this is due to the adjustment of baseline scores of role functioning and mental health and covariables or due to the INA. Therefore, this result should be approached cautiously.

Several factors may help to explain the observed lack of change in (health-related) quality of life and well-being. The social and political climate in which the INA was initiated may have contributed to these results. During this period, the municipality of Rotterdam implemented an array of policy changes—mainly in home care—and used competitive tender practices to appoint (new) health and social care providers. As described elsewhere [20] the rate and complexity of these reforms were detrimental to established community relationships and generated high levels of mutual distrust and insecurity among INA partners, including older people. Dynamic environments often hamper the ability to innovate and create learning environments [21] and multicomponent interventions are particularly sensitive to contextual factors [22]. The achievement of multilevel alignment across professional, organizational, and policy borders through INA implementation may require more time, continuity, and broad commitment throughout all levels (i.e., micro-, meso-, and macrolevels) [7, 23].

In addition to being distracted by the dynamic environment from developing and optimizing the intervention, community workers struggled to find innovative ways to support older people and lacked helpful support tools [20]. Paradoxically, the project team’s provision of ample professional autonomy paralyzed INA community workers in their search for innovative working methods. For example, community workers were expected to rely on informal support before seeking professional support; however, due to barriers to informal support provision and receipt [24], they often relied on conventional support organization techniques [20]. Given the complexity of evolution toward innovative norms and practices, the 1-year study period may have been insufficient to capture intervention optimization and to detect effects on older people’s health and well-being [25].

Previous research has also demonstrated that integrated care initiatives regularly fail to achieve expected outcomes. Several recent reviews of integrated care programs have revealed unconvincing and inconclusive effects on care outcomes [26–30]. Although these reviews focused on “conventional” components of integrated care, such as the integration of health and social care services and the use of multidisciplinary teams and preventive home visits, they did highlight the complexity of integrated care and support initiatives. Barriers associated with meeting the complex and varied needs of frail older people and those related to contexts characterized by competing economic and social pressures challenge the effectiveness of initiatives. These initiatives will not necessarily fail to meet expectations, but we are still in the process of learning which types of intervention are appropriate in different contexts and for which recipients [30].

This study has several limitations. Although we matched intervention and control participants, the groups showed notable baseline differences. Differences in age, educational level, income, living situation, morbidity, and many outcome measures [well-being and three of five (health-related) quality of life subscales] favored the control group. Adjustment for baseline measures may not have been sufficient to account for unobserved differences. The suitability of the TFI as a matching tool is also uncertain; although it identifies frail older people and has shown predictive validity for disability and quality of life [31] it may not cover all aspects of frailty and thus should not be used in isolation. Furthermore, TFI administration differed between groups; it was self-administered in the control group and administered by community workers during home visits to intervention participants. INA community workers indicated that some older people appeared to mask the severity of their conditions in their presence (e.g., due to fear of institutionalization). Future research is required to establish whether TFI scores vary according to the method of administration.

Moreover, the outcome measures used in this study may have been to distal and global to detect group differences. To allow a more nuanced understanding of the intervention’s effectiveness, future research may also take into account more immediate measures that depict specific aspects of (health-related) quality of life and well-being, such as social participation, loneliness and social well-being.

The use of different recruitment methods may also have contributed to baseline differences between groups [32, 33]. Community workers recruited intervention participants, whereas a random sample of control subjects was recruited by mail and telephone. Unlike in many other community-based integrated care interventions, which rely on systematic visitation of older people listed in GPs’ registries, INA community workers depended on professionals and community members to identify frail older people. This difference in approaches may have affected the composition of the intervention group. Furthermore, older people’s agreement to participate at community workers’ requests may have been based on personal or social desirability motivations. Postal questionnaires, such as that used for control group recruitment, may be especially sensitive to selective non-response, leading to overrepresentation of willing individuals who feel physically and cognitively capable of participation [34]. This possibility is supported by the lower response rates from less-advantaged neighborhoods in our sample.

## Conclusions

This thorough study, which included three measurements and a control group, demonstrated that the INA does not (yet) meet expectations. Given the complexity of the INA, the 1-year study period may have been too short for intervention optimization and detection of effects on outcomes in older people. Complex interventions such as the INA may require a “bedding-in” period before extensive evaluation of processes and outcomes is appropriate [35]. Our findings also indicate the need to further improve and refine such programs before large-scale implementation. Although current demands require decisiveness, we must remain critical and carefully determine which interventions are most appropriate, considering local contexts and beneficiaries.
